# Distance to hospital is not a risk factor for emergency colon cancer surgery

**DOI:** 10.1007/s00384-018-3074-y

**Published:** 2018-05-24

**Authors:** Niillas Blind, Karin Strigård, Ulf Gunnarsson, Fredrik Brännström

**Affiliations:** Department of Surgical and Perioperative Sciences, Umeå University, SE-901 88 Umeå, Sweden

**Keywords:** Colon cancer, Emergency surgery, Distance, Rural

## Abstract

**Purpose:**

The purpose of this study is to see if the distance to a hospital performing colon cancer surgery is a risk factor for emergency surgical intervention and to determine the variability between defined but demographically divergent catchment areas.

**Methods:**

Data on patients living in Västerbotten County who underwent colon cancer surgery between 2007 and 2010 were extracted from the Swedish Colorectal Cancer Register (SCRCR). Of the 436 registrations matching these criteria, 380 patients were used in the analysis, and their distance to the nearest hospital providing care for colorectal cancer (CRC) was estimated using Google Maps™. The correlations between the risk for emergency surgery and the distance to a hospital, gender, age, income level and hospital catchment area were analysed in uni- and multivariate models.

**Results:**

Distance to the nearest hospital had no significant effect on the proportion of emergency operations for colon cancer. There was significant variability in risk for emergency surgery between hospital catchment areas, where the catchment areas of the university hospital and the most rural hospital had a higher proportion than the other local hospital catchment area (OR, 2.00 (*p* = 0.038) and OR, 2.97 (*p* = 0.005)). These results were still significant when analysed with multivariate logistic regression (OR, 2.13 (*p* = 0.026) and OR, 3.05 (*p* = 0.013)).

**Conclusion:**

Distance to a hospital performing colon cancer surgery had no effect on the proportion of emergency surgeries. However, a variability between defined catchment areas was seen. Future studies will focus on possible factors behind this variability.

## Introduction

In Sweden, the proportion of all colon cancer surgery performed as an emergency is 21.5% [1]. Patients having emergency surgery for colon cancer have worse short- and long-term survival rates than elective cases [2, 3]. The most common reasons for emergency surgery are obstruction, perforation and bleeding [4], and tumour stage is often more advanced [5]. Various factors can prolong the time it takes to come to a diagnosis of colorectal cancer (CRC), including delay on the part of the patient and of their doctor [6, 7]. Persons living alone, for example, tend to seek healthcare at a later stage for symptoms suggestive of CRC [8], and socioeconomic status also has an impact on delay of diagnosis and proportion of colon cancer surgery performed as an emergency [9, 10]. Previous studies on survival in colon cancer have found lower survival rates among patients living in rural areas [11].

The county of Västerbotten in northern Sweden has 263,000 inhabitants [12] divided between 15 municipalities; the majority residing in two towns on the east coast. The municipalities inland are smaller and have a longer distance to travel to a hospital performing colorectal cancer surgery. One of the coastal towns has a university hospital and the other a local hospital. There is also a local hospital in the sparsely populated western part of the county. All three hospitals performed elective and emergency CRC surgery during the study period. This situation with three hospitals covering well-defined rural and urban areas makes Västerbotten a suitable model for investigating the relationship between demography and geography in the treatment of CRC. All patients undergoing surgery for colon cancer in Sweden are reported to the Swedish Colorectal Cancer Register (SCRCR) having a completeness of 99.5%. Operations are classified by the surgeon as either emergency or elective. Emergency surgery is defined in the SCRCR as a procedure performed for medical reasons during an unplanned hospital admission.

Most healthcare providers in Sweden come under the national healthcare service, and this is especially true for cancer care. Hospitals have strict catchment areas based on county borders, and it is uncommon for patients to receive CRC healthcare in hospitals outside their own catchment area [13].

There has been a trend towards centralisation of CRC care based on the small differences in outcome reported between low- and high-volume surgeons and hospitals [14]. However, little is known about the impact of centralisation itself. One effect of centralisation could be that the longer distance that must be travelled to hospitals performing CRC surgery, increases the risk for emergency presentation of colon cancer.

The aim of this study was to see if a longer distance to a hospital providing surgical care for CRC is a risk factor for emergency surgery and to determine the variability of percentage emergency surgery between geographically defined catchment areas.

## Method

Data on all patients who had undergone surgery for colon cancer in Västerbotten County 2007–2010 were retrieved from the SCRCR.

The address of each patient was obtained from the patient’s hospital records. Google Maps™ was used to estimate the distance from each patient’s home to the nearest hospital providing surgical care for CRC. Since the aim was to see if distance to a hospital providing CRC surgery was a risk factor for emergency colon cancer surgery, the distance from the patient address to the nearest such hospital was used also in the rare cases were the surgery actually was performed at another hospital.

Three hospitals were included: the university hospital on the coast (hospital A); the local hospital on the coast (hospital B); and the rural local hospital inland (hospital C). There are differences in population density between these hospitals, with hospital A having 15.7, hospital B 8.9, and hospital C 1.0 inhabitants/km^2^. Data were also collected regarding average income, age, gender and municipality population density.

To analyse the role of income level, the population was divided into two groups based on the mean income of the municipality of residence, since individual income details were not available. The two largest municipalities were also those with the highest mean incomes and thus formed the high-income group. The other 13 smaller municipalities formed the low-income group.

The population was further divided into three groups based on the population density of the municipality of residence. The largest municipality constituted a group on its own, two intermediary populated municipalities formed the second group and the remaining 12 formed the most sparsely populated group.

The population was divided into three groups according to age; the youngest quartile and the oldest quartile groups, with the two intermediary quartiles forming the reference group. The reason for this was that the relationship between age and the risk for emergency surgery was expected to be non-linear with the youngest and/or the oldest age groups having divergent values. Since the relationship between distance to the hospital and risk for emergency surgery was not known, both a linear assumption (linear regression) and an arbitrary division into four groups based on quartiles (logistic regression) were tested.

To ensure that data on emergency priority were valid, 47 random records were checked by three colorectal surgeons, blinded and separated from each other, to see if the priority in the records matched the priority given in the register.

In the cases where data on priority were missing, a priority assessment was made retrospectively, based on the patient’s records.

## Statistics

Uni- and multivariate linear and logistic regressions were used. In the multivariate analyses, all variables were entered at the same time (force entry). Since the parameters describing income and catchment areas were both based on municipalities, they were not considered independent from each other and therefore not applied in the same multivariate models. All analyses were performed using STATA version 13.1 (StataCorp LP, College Station, TX, USA).

## Ethics

Ethical approval for this study was obtained from the Regional Ethics Committee in Umeå (Dnr 2015/143-31).

## Results

**Fig. 1:**
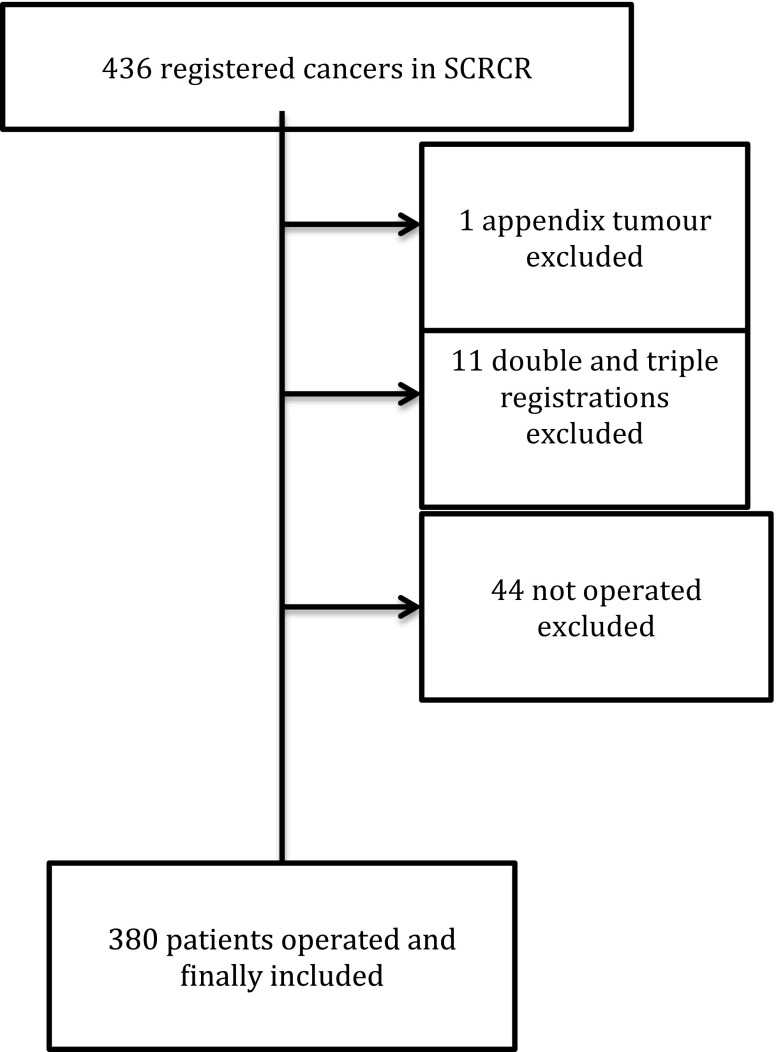
Flow chart of patients retrieved from the SCRCR. Of the 380 patients finally included, 365 underwent resection surgery, 2 received a stent and 3 a stoma as emergency procedure, and the remaining had different surgical procedures

There were 436 registrations on 425 individuals from the defined time period in the SCRCR. Only the first operation was included for patients who had had surgery more than once. After exclusion of double and triple registrations, patients not operated on and a tumour of the appendix, 380 procures remained for analysis (Fig. 1).

Two of these three hundred eighty patients received a stent as a bridge to surgery, and three patients received a stoma before resection. In eight cases, priority was judged using the patient’s records because data were missing in the register.

In one of the forty-seven cases checked (2%) by the three blinded surgeons, the priority (elective/emergency) noted in the records was considered not to match that in the register.

The mean age of the patients included was 72 years, and 53% were female. The quartiles for age were 0–66, 67–72, 73–78 and 79–88 years. The quartiles for distance were 0–3.49, 3.5–13.79, 13.8–51.19 and 51.2–232 km.

**Fig. 2:**
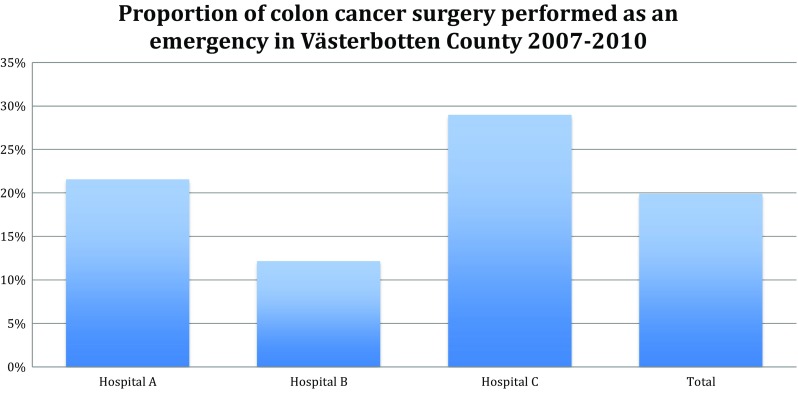
Proportion of colon cancer surgery performed as an emergency in three different catchment areas in Västerbotten County. Hospital A, Umeå (University hospital); hospital B, Skellefteå (Local hospital); hospital C, Lycksele (Local hospital in rural area)

The mean distance to a hospital providing CRC surgery was 32.6 km (range, 0.1–231.6 km). Of the patients included, 77 (19.89%) had emergency surgery. In the 4th distance quartile, the mean distance was 93 km, and in the > 90% group, the mean distance was 130 km. The only significant difference in prevalence of emergency surgery was related to catchment area (Fig. 2). No difference was found concerning distance to hospital (Table 1). The results of the multivariate linear regression models did not reveal any relevant difference from the univariate models. There was no significant difference in the risk for emergency surgery between the high- and low-income groups (> 23,439 and < 22,856 euro/year) (Table 2).

**Table 1:** Distance to hospital for different patient groups operated for colon cancer in Västerbotten County

Number		Mean distance (km)	*p* value
Priority			
304/380	Elective	32.63	Ref
76/380	Emergency	37.01	0.433
Estimation of surgeon			
318/380	Curative surgery	33.82	Ref
62/380	Palliative surgery	31.58	0.748
Type of operation			
365/380	Resection surgery	33.68	Ref
15/380	Other surgery	29.84	0.722
Age			
96/380	1st quartile	33.98	0.949
185/380	2nd–3rd quartiles	33.63	Ref
99/380	4th quartile	32.81	0.881
Gender			
205/380	Female	34.22	Ref
175/380	Male	32.67	0.729
Population density			
138/380	High	8.86	Ref
123/380	Average	16.41	0.044
119/380	Low	79.76	0.000
Income			
244/380	High	10.93	Ref
136/380	Low	74.00	0.000

Distance to a hospital performing CRC surgery for different patient groups in the CRC database for Västerbotten County 2007–2010

**Table 2:** Risk factors for emergency surgery for colon cancer in Västerbotten County

Number		Emergency surgery	Univariate			Multivariate		
			Odds ratio	95% CI	*p* value	Odds ratio	95% CI	*p* value
Distance								
91/380	1st quartile	20.8%	1.48	0.70–3.11	0.305	1.47	0.68–3.20	0.332
99/380	2nd quartile	15.2	1.00	Ref	Ref	1.00	Ref	Ref
94/380	3rd quartile	20.2	1.42	0.67–2.99	0.358	1.52	0.71–3.24	0.281
96/380	4th quartile	24.0	1.76	0.86–3.63	0.123	1.26	0.55–2.92	0.582
Distance (90%)								
339//380	< 90th (%)	19.5%	1.00	Ref	Ref			
41/380	> 90th (%)	24.4	1.33	0.62–2.36	0.458			
Age								
96/380	1st quartile	18.8%	0.84	0.44–1.53	0.573	0.79	0.42–1.49	0.467
185/380	2nd–3rd quartiles	21.6	1.00	Ref	Ref	1.00	Ref	Ref
99/380	4th quartile	18.2	0.81	0.43–1.50	0.494	0.79	0.42–1.50	0.478
Gender								
205/380	Female	18.0%	1.00	Ref	Ref	1.00	Ref	Ref
175/380	Male	22.3	1.30	0.79–2.15	0.304	1.35	0.81–2.25	0.256
Population density								
138/380	High	21.0%	1.00	Ref	Ref			
123/380	Average	13.8	0.60	0.31–1.16	0.130			
119/380	Low	25.2	1.27	0.71–2.27	0.426			
Hospital catchment area								
195/380	Hospital A	21.5%	2	1.04–3.85	0.038	2.13	1.09–4.13	0.026
116/380	Hospital B	12.1	1.00	Ref	Ref	1.00	Ref	Ref
69/380	Hospital C	29.0	2.97	1.39–6.38	0.005	3.05	1.27–7.34	0.013
Average income								
244/380	High	17.6%	1.00	Ref	Ref			
136/380	Low	24.3	1.50	0.90–2.50	0.122			

Uni- and multivariate logistic regressions of factors potentially influencing the risk for emergency colon cancer surgery. Age and distance divided in quartiles. Distance to the closest hospital performing CRC surgery was also analysed using a model divided at the 90th percentile

## Discussion

Distance to the nearest hospital performing CRC surgery did not have an impact on the risk for emergency surgery for patients with colon cancer. This indicates that the distance to hospital does not affect the time passed between symptom presentation or diagnosis and surgery. With our current data set, it is not possible to make any absolute conclusions regarding extreme distances. However, we make the assessment that the distances calculated are relevant for European circumstances. An alternative interpretation is that difference in the delay to elective surgery does not affect the risk for emergency surgery. It could be that the biology of the tumour or patient characteristics are more important risk factors for emergency colon cancer surgery.

The small differences in mean distance to hospital found between emergency and electively operated patients strengthens our conclusion that there is no clinically relevant and unbiased correlation between the distance to hospital and the risk for emergency surgery. The non-linearity of differences in prevalence of emergency surgery between the distance quartiles further strengthens this conclusion. It is thus unlikely that our failure to detect an effect of distance on risk for emergency surgery was due to a lack of statistical power.

The difference in proportions of colon cancer surgery performed as an emergency seen in the three hospital catchment areas is more difficult to interpret. It seems unlikely that differences in biological characteristics of tumours or patients could explain a relationship between living in a specific hospital’s catchment area and the risk for emergency surgery. It is more likely that the difference between these catchment areas in delay from onset of symptoms or diagnosis to surgery is the cause. However, since there were only three catchment areas represented in this study, it is not possible to draw any firm conclusions regarding the cause of variability. Nevertheless, the large variability in prevalence of emergency surgery between the three catchment areas is an interesting finding that requires further investigation. The lowest percentage emergency surgery was at one of the local hospitals (12%). Should it be possible to attain a similar figure for the general population of CRC patients, a considerable impact on the overall prognosis of colon cancer would be achieved.

Numerous studies on the relationship between duration of symptoms and prognosis have failed to indicate a worse prognosis for those with long delay between onset of symptoms and treatment [15]; in fact, the opposite seems to be the case [16, 17]. Patients with long distances to travel to a hospital performing CRC surgery also tend to show up with more advanced tumour stages [18]. The main reason for not seeking medical advice for symptoms of colon cancer is because the patient does not believe the situation is serious and that the problem will disappear spontaneously [19, 20]. The distance to the nearest hospital is unlikely to influence this behaviour since the patient seeks advice at the local healthcare centre first, and this is usually much closer to home than the hospital performing CRC surgery. Subsequent referral for further investigation with coloscopy, CT etc. is probably more dependent on the general practitioner and local healthcare logistics than on the patient.

If we are to reduce the numbers of emergency surgical procedures for CRC, then we must detect cancer before it gives rise to symptoms. Screening is probably one of the most effective ways of decreasing the prevalence of emergency surgery for colon cancer. It has been shown that distance to the healthcare provider is a barrier when screening for breast cancer [21], and this may also apply to colon cancer screening. If this should be the case, then distance to the screening hospital could have an impact on the risk for emergency surgery in a population where screening is well established.

A limitation of this study is the relatively small number of patients and that only one county was included. The main advantage is that the study was population based and includes all patients who underwent resection surgery in a well-defined area without exclusions. The catchment areas of the three hospitals are strictly defined and healthcare is government funded. There are very few exceptions where private means or insurance policies play a significant role in financing healthcare in this area. Furthermore, distances travelled were measured from the patient’s home at the time of their operation to the nearest hospital providing CRC surgery and not on estimations based on zip code centroids.

This study analysed income groups at municipality level only. Since there was a fairly large but non-significant difference in the risk for emergency surgery between the income groups, it would be of interest to investigate the impact of income at the individual level in a larger study population.

## Conclusion

Distance to a hospital providing CRC surgery is not a risk factor for emergency colon cancer surgery. However, when comparing the hospital catchment area with the lowest proportion of CRC procedures performed as an emergency, with the other two hospital catchment areas in this study, the difference in risk was two to threefold. It thus seems likely that there are other factors that determine the risk for emergency colon cancer surgery that we are unable to target with the present dataset and population.
